# Genetic Mapping and Phenotypic Analysis of *GstE14 ^E.4.1^* on Eye and Antennae Development in *Drosophila melanogaster*

**DOI:** 10.17912/micropub.biology.001019

**Published:** 2024-04-13

**Authors:** Lauren Thomson, Hemin P Shah, Victoria Akinwotu Adewale, Alyssa Beise, Camryn Bliayang, Zuzanna Cioch, Mason Craig, Adell Crump, Maya Durdan, Madeleine Espinosa, Kaitlin Feda, Jami Feist, Alexis Fragoso, Genesys Haro, Breanna Hoffman, Paige Horne, Nathan Houha, Shirley Hounnou, Annabel Inman, Daniel Jakobsze, Yolanda Juarez-Morales, Yousuf Khan, Joshua Kohler, Reece Lawlor, Bethany Lieser, Ryan Loitz, Erik Martinez, Alexis Martinez, Michelle Martinez, Brandyn Maza, Brenda Mendoza, Steven Miller, Haniel Mngodo, Sarah O'Shea, Sarah N Piane, Ethan Raivala, Sophie Ruger, Abigail Singer, Jessica E Strand, Alexis Traylor, Asia Wright, Shawn McCabe, Sandesh S Pandit, Kayla Bieser, Paula Croonquist, Elizabeth E Taylor, Jacqueline Wittke-Thompson, Jacob D Kagey, Olivier Devergne

**Affiliations:** 1 Northern Illinois University, DeKalb, Illinois, United States; 2 University of St. Francis, Joliet, Illinois, United States; 3 Anoka-Ramsey Community College, Coon Rapids, Minnesota, United States; 4 Nevada State University, Henderson, Nevada, United States; 5 Universty of Detroit Mercy, Detroit, Michigan, United States

## Abstract

Genetic screens are valuable for identifying novel genes involved in the regulation of developmental processes. To identify genes associated with cell growth regulation in *Drosophila melanogaster* , a mutagenesis screen was performed. Undergraduate students participating in Fly-CURE phenotypically characterized the *E.4.1* mutant which is associated with rough eyes and antennae overgrowth. Following complementation analysis and subsequent genomic sequencing, *E.4.1* was identified as a novel mutant allele of *GstE14* , a gene involved in ecdysone biosynthesis important for the timing of developmental events. The abnormal eye and antenna phenotypes observed resulting from the loss of *GstE14* suggest its role in tissue growth.

**Figure 1. Characterization of the lethal GstE14 E.4.1 mutation by phenotypic analysis, complementation mapping, and genetic sequencing:**
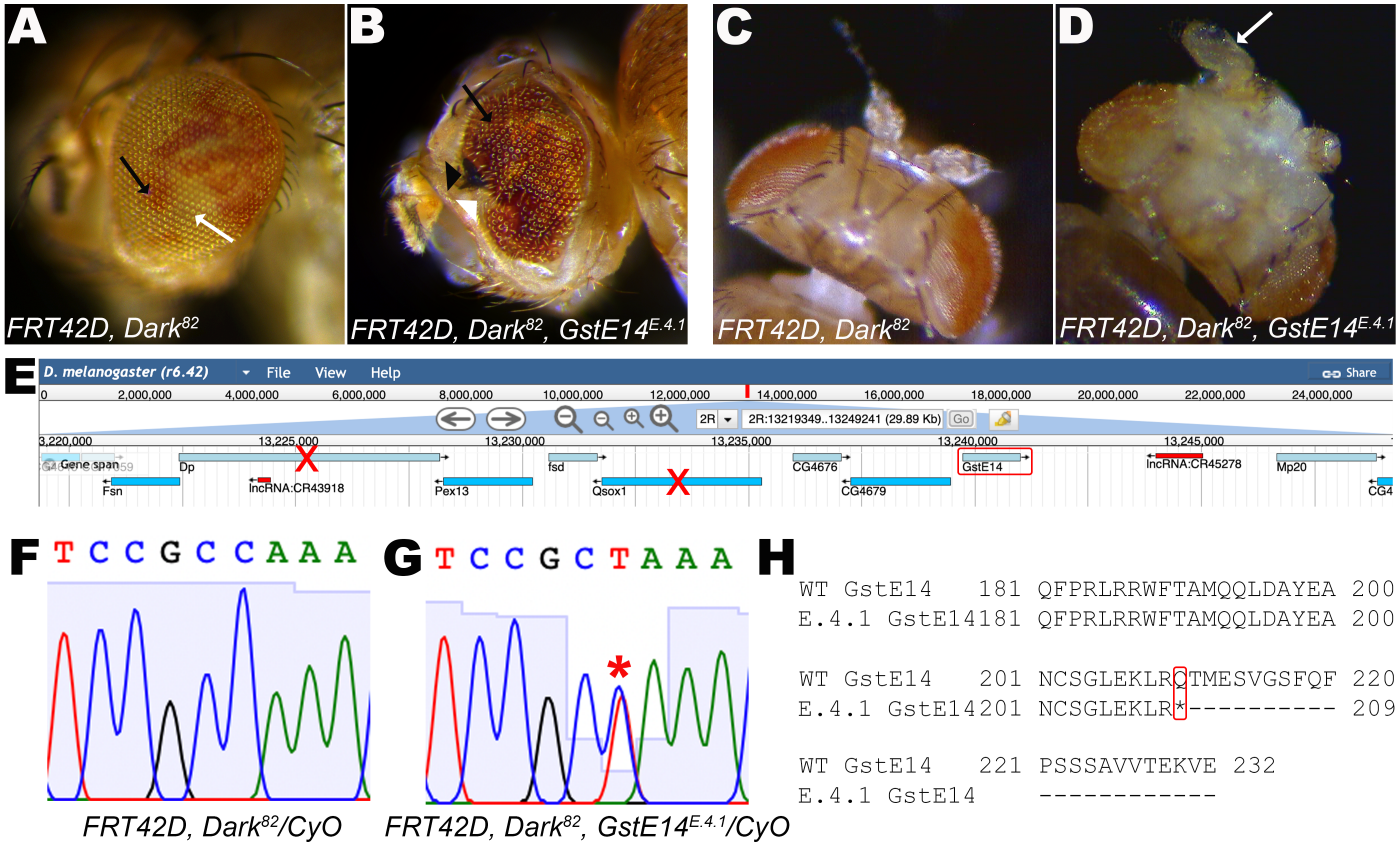
(A) *FRT42D, Dark ^82^* control mosaic eye (B) and *FRT42D, Dark ^82^ , GstE14 ^E.4.1^* mutant mosaic eye showing white (wildtype, white arrow) and red (mutant, black arrow) pigmentation as a result of FRT/FLP mitotic recombination during development. (B) Overgrowth of mutant tissue in genotype *FRT42D, Dark ^82^ , GstE14 ^E.4.1^* is observed as clusters of red pigmentation, in addition to mutant clones displaying necrotic tissue (black arrowhead) in mosaic eye and rough eye phenotype with disorganized ommatidial arrangement (white arrowhead). (C) *FRT42D, Dark ^82^* control fly shows wildtype antennae. (D) *FRT42D, Dark ^82^ , GstE14 ^E.4.1^* mutants exhibit antenna overgrowth (arrow). (E) The narrowest region in which *E.4.1* failed to complement in the genomic region 2R:13,219,130..13,249,241 (Image adapted from JBrowse on FlyBase). (F-G) Sanger sequence analysis of wildtype *GstE14* and mutant *GstE14 ^E.4.1^* reveals a heterozygous peak of C → T at 2R:13,240,692. (H) Alignment of amino acids of control *FRT42D, Dark ^82^* and mutant *FRT42D, Dark ^82^ , GstE14 ^E.4.1^* sequence show the presence of a nonsense mutation at amino acid 210 (Gln → Stop) in the *GstE14 ^E.4.1^* mutant resulting in a truncated protein missing the last twenty two amino acids in the C-terminal region of GstE14.

## Description

To identify novel genes involved in the regulation of cell growth processes in the developing *Drosophila melanogaster* eye, an ethyl methanesulfonate (EMS) mutagenesis screen was carried out utilizing the FLP/FRT recombination system on chromosome 2R (Kagey et al., 2012) . For this screen, the EMS concentration used was at levels that have been shown to result in an average of one lethal hit per chromosome arm. The mutations generated in this screen are homozygous lethal, so the FLP/FRT recombination system was used to generate mutant cell clones in the eye for phenotypic characterization while maintaining heterozygosity in the organism to prevent mortality. Although the *Drosophila* eye is not critical for survival, apoptotic pathways may be triggered in mutant cells so that an overgrowth phenotype associated with the mutation may not be observed. Therefore, apoptosis was prevented in mutant clones by the utilization of *Dark ^82^* , a null allele of *Death-associated* *APAF-1 related killer* ( *Dark* ), on chromosome 2R distal to cytological site 42D (Akdemir et al., 2006; Mills et al., 2006) . Blocked apoptosis allows for an overgrowth phenotype to progress to an observable state. The *Dark ^82^* mutant allele is due to the insertion of a *mini-white* P-element, allowing the identification of mutant clones by the presence of red pigmentation after mitotic recombination. Here we present the phenotypic characterization and the genetic mapping of the *E.4.1* mutant line isolated in this screen.

To analyze the phenotype of *E.4.1* , male flies of the mutant genotype ( *FRT42D* , *Dark ^82^* , *E.4.1* / *CyO* ) and of the control genotype ( *FRT42D* , *Dark ^82^* / *CyO* ) were crossed with virgin females of genotype ( *ey-Flp; FRT42D)* . Since the eyeless ( *ey* ) promoter is active in the eyes, the restricted mitotic recombination leads to the generation of a mosaic eye containing homozygous *E.4.1* mutant cells (red) and WT cells (white). The resulting F1 generation of the mutant cross was compared to that of the control cross to identify differences in tissue growth in the mosaic eye and morphological abnormalities. The quantified data showed an average of 56% red ( Figure 1A, black arrow ) to 44% white ( Figure 1A, white arrow ) tissue in the mosaic eyes of the control *FRT42D, Dark ^82^* flies, with no signs of overgrowth of eyes or surrounding tissue (n=98) Figure 1A, 1C). However, *FRT42D, Dark ^82^* , *E.4.1* mutant clones ( Figure 1B, 1D) displayed overgrowth of mutant (red) eye tissue ( Figure 1B, black arrow ) and the generation of rough eyes manifesting as red clusters lacking precise ommatidial arrangement ( Figure 1B, white arrowhead ). Quantification of mosaic eye tissue resulted in an average of 95% red to 5% white tissue (n=161). Furthermore, heterozygous (orange) tissue was also present in the mutant eyes, with an average of 15% orange to 85% red (mutant) tissue (n=161). Additional abnormalities were observed, such as the presence of likely necrotic tissue on the compound eye (21% of *E.4.1* mutant eyes present likely necrotic tissue, n=223) ( Figure 1B, black arrowhead ) and the antenna. We also observed enlarged antennae in E.4.1 mosaic adult fly compared to the control mosaic fly ( Figure 1D, arrow compared to Figure 1C).

In parallel, a complementation analysis was performed to narrow down the genomic location of the *E.4.1* mutation and identify the gene affected by the *E.4.1* mutation, using the Bloomington 2R Deficiency Kit (BDSC Df(2R) kit) with deletions of known endpoints on chromosome 2R distal to the FRT42D site (Cook et al., 2012) . Virgin females of genotype *FRT42D* , *Dark ^82^* , *E.4.1* / *CyO* were crossed with males of the genotype Df(2R)/CyO. The F1 progeny from each cross were examined for the presence or absence of straight-wing flies, where the presence of only curly-wing flies indicates a failure to complement the mutation. In the first round of mapping, *E.4.1* failed to complement with deficiency lines *Df(2R)CX1* and *Df(2R)BSC273* (Table 1) whereas, deficiency lines *Df(2R)Exel8057* and *Df(2R)BSC274* complemented *E.4.1.* Thus, identifying the region 2R:13,219,349..13,430,464 as the putative chromosomal location for the E.4.1 mutation. Additionally, *Df(2R)BSC331* failed to complement with *E.4.1.* However, the genomic region covered by *Df(2R)BSC331* was excluded from the possible genomic location for the *E.4.1* mutation since it contains *Dark* , and served as a positive control for the complementation mapping. A second round of complementation analysis was performed within the region 2R:13,219,349..13,430,464 to further define the genomic location of *E.4.1* . Of these, *Df(2R)Exel7124* and *Df(2R)BSC272* failed to complement, resulting in 2R:13,219,130..13,249,241 as the smallest region that failed to complement the *E.4.1* mutation ( Figure 1E ).

Next, a complementation analysis was performed for *Dp* and *Qsox1* , the only two of the candidate genes for which homozygous lethal mutants were available at *Drosophila* stock centers. They both complemented with *E.4.1* indicating that this mutation does not affect these two genes.

Then, to identify the gene affected by the *E.4.1* mutation, we sequenced the remaining genes. To do so, genomic DNA was isolated from *FRT42D* , *Dark ^82^* , *E.4.1* / *CyO* mutant and FRT42D, *Dark ^82^* / *CyO* control fly lines, and primers were designed to perform PCR amplification and Sanger sequencing. Subsequent sequence analysis showed a single nucleotide change (C→T) in the *E.4.1* mutant line compared to the control at 2R:13,240,692. This mutation was independently confirmed by whole genome sequencing of the *E.4.1* mutant line (Bieser, unpublished results). This mutation affects the coding region of the *Glutathione S transferase E14* (GstE14) resulting in a premature stop codon at amino acid 210 (Gln → Stop) ( Figure 1H ). Overall, our data suggest that the potential loss of functional GstE14 leads to tissue overgrowth.

Following complementation and sequence analysis, we conclude that *E.4.1* is a novel mutant allele of *GstE14* ( *GstE14 ^E.4.1^* ) that truncates the resulting protein due to a nonsense mutation ( Figure 1H ). *GstE14 ,* also referred to as *noppera-bo* ( *nobo* ), encodes a glutathione S-transferase, an ecdysteroidogenic enzyme that is suggested to be crucial in the biosynthesis of ecdysone (Enya et al., 2014) . Ecdysone is a major insect ecdysteroid synthesized in the prothoracic gland from exogenous sterols, such as cholesterol, and its release ensures that metamorphosis and molting occur at the appropriate time during morphogenesis (Gilbert et al., 2002; Niwa & Niwa, 2014) . *GstE14* has been previously characterized as a novel Halloween gene, as its loss-of-function results in phenotypes indicative of low ecdysone production. However, functional disruption of *GstE14* additionally results in atypical accumulation of cholesterol in the prothoracic gland, suggesting that its function is important in the metabolism and/or transport of cholesterol (Enya et al., 2014) .

When the biosynthesis and release of ecdysone are efficiently regulated, it directly influences the differentiation of larval imaginal disc tissues, including the eye imaginal disc, to form the adult structures, as well as signals the eradication of larval tissues that are no longer required in the adult fly through steroid-driven apoptotic pathways (Brennan et al., 1998; Cranna & Quinn, 2009) . Interferences with these mechanisms of imaginal disc differentiation or apoptotic pathways leading to the final adult structure of *Drosophila melanogaster* are consistent with the *E.4.1* mutant phenotype observed in and around the eye.

Though *GstE14* has no direct human ortholog, human glutathione S-transferases are a topic of interest in the field of oncology and chemotherapeutic treatments. *Drosophila melanogaster* *GstE14* resides in the epsilon class of GSTs, a class unique to arthropods (Škerlová et al., 2020). Evolutionarily, this class of GSTs likely emerged in these species as an adaptation to the environment in insects specifically (Gonis et al., 2022) . However, developing an understanding of the GST protein family as a whole may be important for the treatment of many cancers. It has been found that GSTs are overexpressed in cancer cells, suggesting their potential role in metastasis (Singh & Reindl, 2021) . Since GSTs play a role in regulating redox homeostasis within the body's cells, these enzymes are able to detoxify chemotherapeutic agents, contributing to resistance against them (Shen et al., 1997) . While *GstE14* 's complete role in ecdysone biosynthesis and its direct substrates remains unknown, further experiments revealing these properties could aid in a greater understanding of the role of Glutathione S-transferases in human disease.

**Table 1.** Results of complementation analysis with deficiency lines of chromosome 2R and individual mutant alleles when scored for complementation against homozygous lethal mutant *E.4.1* .

**Table:** 

Bloomington Stock Center 2R Deficiency Kit			
**Deficiency Stock**	**BDSC Stock #**	**Chromosomal Deletion**	** Complementation Result with *E.4.1***
*Df(2R)CX1*	442	2R:12,700,421..14,091,140	Fail to complement
*Df(2R)Exel8057*	7871	2R:13,034,847..13,219,349	Complement
*Df(2R)BSC273*	23169	2R:13,159,579..13,502,150	Fail to complement
*Df(2R)BSC274*	23170	2R:13,430,464..13,593,272	Complement
*Df(2R)BSC331*	24356	2R:16,869,330..17,139,923	Fail to complement
Additional Deficiency Lines			
**Deficiency Stock**	**BDSC Stock #**	**Chromosomal Deletion**	** Complementation Result with *E.4.1***
*Df(2R)Exel7124*	7872	2R:13,219,130..13,281,253	Fail to complement
*Df(2R)BSC272*	23168	2R:13,219,130..13,249,241	Fail to complement
Mutant Alleles of Individual Genes			
**Gene**	**BDSC Stock #**	**Allele**	** Complementation Result with *E.4.1***
*Dp*	5553	*Dp ^49Fk-1^*	Complement
*Qsox1*	77650	*Qsox1 ^4037-G4^*	Complement

## Reagents

*w ^-^ ,FRT42D, Dark ^82^ / CyO* (Akdemir et al. 2006)

*w ^-^ ,FRT42D, Dark ^82^ , GstE14 ^E.4.1^ / CyO* (this manuscript)

*w ^-^ , ey-FLP; FRT42D* (BDSC 5616)

Bloomington *Drosophila* Stock Center 2R Deficiency Kit (Cook et al. 2012)

*w ^1118^ ;Df(2R)Exel7124/CyO* (BDSC 7872)

*w ^1118^ ; Df(2R)BSC272/CyO* (BDSC 23168)

*b ^1^ Dp ^49Fk-1^ c ^1^ /SM5* (BDSC 5553)

*w ^1118^ ; PBac{w ^+mC^ =IT.GAL4}Qsox ^4037-G4^ /CyO* (BDSC 77650)

*GstE14* forward primer 1: 5' AGTTACTGATCGACTTTCAAGGCGTTC 3'

*GstE14* forward primer 2: 5' GCACGCAGAACGGATGAAGG 3'

*GstE14* reverse primer: 5' CTGTCATGAATTTCTATTGGCGAGTCATTA 3'
